# Exposure to Bisphenol A and Its Analogs among Thai School-Age Children

**DOI:** 10.3390/toxics11090761

**Published:** 2023-09-08

**Authors:** Nattakarn Numsriskulrat, Thanawan Teeranathada, Chansuda Bongsebandhu-Phubhakdi, Suphab Aroonparkmongkol, Kyungho Choi, Vichit Supornsilchai

**Affiliations:** 1 Division of Academic Affairs, Faculty of Medicine, Chulalongkorn University, Bangkok 10330, Thailand; nnk_natty@docchula.com; 2 Division of Pediatric Endocrinology, Department of Pediatrics, Faculty of Medicine, Chulalongkorn University, Bangkok 10330, Thailand; b.teeranathada@hotmail.com (T.T.); chansuda.b@gmail.com (C.B.-P.); 3 Division of Pediatric Endocrinology, Department of Pediatrics, King Chulalongkorn Memorial Hospital, Bangkok 10330, Thailand; aroonpark@yahoo.com; 4 Department of Environmental Health Sciences, Graduate School of Public Health, Seoul National University, Seoul 08826, Republic of Korea; kyungho@snu.ac.kr

**Keywords:** bisphenol A, bisphenol F, bisphenol S, liquid chromatography, tandem mass spectrometry, children, exposure

## Abstract

Bisphenol F (BPF) and bisphenol S (BPS) have become popular substitutes for bisphenol A (BPA) in the plastic industry due to concerns over BPA’s adverse effects. However, there is limited information on children’s exposure to these chemicals. This study aims to assess the extent of BPA, BPF, and BPS exposure and determine factors that influence such exposure. A group of Thai children (age 6–13 years, N = 358) were recruited between October 2019 and 2020. Two first-morning voids were collected one week apart. Demographic and exposure-related information was gathered. Urinary concentrations of bisphenols were analyzed by liquid chromatography and tandem mass spectrometry. Correlation between bisphenol concentrations with age, body weight, and sources of bisphenol exposure, was determined using generalized estimating equations with linear model. BPA, BPF, and BPS were detected at 79.6%, 31.0%, and 16.8%, with geometric mean (GM) concentrations of 1.41, 0.013, and 0.014 ng/mL, respectively. Younger children aged <10 years exhibited 1.3–1.6 times higher GM levels of all bisphenols compared to older children. Exposure to food stored in plastic containers was associated with higher levels of BPF and BPS. In conclusion, BPA was the most frequently detected bisphenol in urine samples from Thai children, followed by BPF and BPS.

## 1. Introduction

Bisphenol A (BPA), a well-recognized ‘Endocrine Disrupting Chemicals (EDC)’ previously used in the plastic industry, has been regulated in many countries due to its association with harmful effects on the human body, especially the endocrine system, including metabolic problems, obesity, precocious puberty, and reproductive dysfunction [1,2,3,4,5,6]. Apart from endocrinopathy, high maternal BPA exposure is also associated with cardiovascular diseases [7] and behavioral problems in offspring [8], reflecting the potential influences on the developmental programming [7] and broader impact of transgenerational BPA toxicity. Because of these unhealthy effects, alternative bisphenol analogs, such as bisphenol F (BPF) and bisphenol S (BPS), are now being used as BPA substitutes [9]. These EDC can be found in cleaning products, thermal paper, coatings, dental sealants, personal care products, canned food, food packaging, and house dust [10,11]. After exposure from ingestion, inhalation, or topical absorption, bisphenols are metabolized by hepatic uridine 5′-diphosphate-glucuronosyltransferases to the corresponding glucuronides and subsequently eliminated in urine [12] within 24 h for BPA [13] and 48 h for BPS [14]. Therefore, urinary levels of these substances are representative biomarkers of human exposure to bisphenols.

BPF and BPS have been extensively studied in recent research and have been found to share structural similarities with BPA, which could potentially lead to comparable endocrine-disrupting effects both in vitro and in vivo [11]. In animal models, these compounds were observed to induce reproductive dysfunction, metabolic issues, alterations in thyroid hormones levels, and behavioral changes [15]. In humans, urinary levels of BPF and BPS were associated with the free androgen index in female adolescents [16]. Similar to BPA, BPS has also been demonstrated to undergo transplacental transfer to the human fetus, as it was detected in samples of umbilical cord blood, placenta, and amniotic fluid [17]. Changes in hematologic parameters and anemia were also observed in pregnant women with high BPF and BPS levels [18,19]. However, research on the adverse effects of BPF and BPS on the health of the developing fetus and children is ongoing. Different natural products, such as plant extracts and natural compounds, have also been investigated for their potential to alleviate the adverse effects of bisphenols, but have turned out to be ineffective to counteract bisphenol-induced toxicity [20]. 

According to recent studies [9,10], exposure to BPA has decreased worldwide due to regulations and the prohibition of its use. However, a worrying pattern emerges as detection rates for BPF and BPS increase, suggesting their widespread presence. The National Health and Nutrition Examination Survey (NHANES) conducted in 2013–2014 showed BPA, BPS, and BPF detection rate of 95.7%, 89.4%, and 66.5%, respectively, of randomly selected urine samples in adults and children [21]. Similarly, a study conducted in European adolescents as part of the Flemish Environment and Health Study (FLEHS IV, 2016–2020) found detection frequencies of over 50% for BPA and its alternatives, BPF, and BPS [22]. In several Asian countries, including China, India, Korea, Kuwait, Malaysia, and Vietnam, a human biomonitoring study reported varying detection frequencies for urinary BPS, ranging from 42 to 91% [21]. Our previous data in 2013 reported that 75.3% of Thai young people aged 3 to 18 years have frequent exposure to BPA [23]. Currently, information on the frequency of exposure to bisphenol in Thai children is lacking. Understanding the current levels of exposure to BPA, BPF, and BPS among children is important, as they are more vulnerable to exposure to EDCs [21,24]. 

This study aimed to determine the prevalence of BPA, BPF, and BPS exposure in Thai school-age children, and to identify factors that influence the urinary concentration of these chemicals. The result of this study can help understand the status of the exposure and develop mitigation measures for major bisphenols among children of Thailand. 

## 2. Materials and Methods

Between October 2019 and October 2020, we enrolled all students from grade 1 to grade 6 in an elementary school in Bangkok, Thailand. This school represented Thai children because it aligned with several key criteria. First, it is in the capital city, which allows us to gather data from a diverse group of middle-class children who may be more exposed to various environmental factors. Additionally, the chosen age group is relevant to the objectives of our study. Moreover, the location of the school, not being near industrial areas, helps minimize potential confounding variables that could affect our research findings. 

The sample size was calculated from the primary objective, using the formula:

$$\mathrm{Sample}\;\mathrm{size}=\frac{Z_{\alpha /2}^{2}\cdot p(1-p)}{d^{2}}$$

$Z_{\alpha /2}^{2}$= 1.96; standard normal variation at 5% type 1 error (*p* < 0.05);

*p* = 0.75; proportion from the previous study [23];

*d* = 0.05; precision;

The sample size will be at least 289 children.

Excluding children with liver or kidney diseases, our study included a total of 358 participants aged between 6 and 13 years. All participating children were measured for height, weight, and waist circumference. Two spot morning urine samples were collected one week apart and stored in cylindrical bisphenol-free containers in a refrigerator at −20 °C until analysis. Since bisphenols have a short half-life of approximately 7 h and are eliminated in urine within 1–2 days after exposure [13,14], changes in exposure patterns could contribute to the observed differences in detection rates and levels between the two urine samples. In this study, we used the average urinary bisphenol concentration so that the data will better reflect true exposure levels to BP among Thai children. The validated questionnaire [23] was conducted with the help of their parents or guardians and asked for socioeconomic status, housing conditions, cosmetic use, consumption of canned food and beverages, and use of plastic food containers. Age- and sex-specific body mass index (BMI) z-scores were calculated using the WHO growth reference standard. All participants were classified into 4 groups: underweight, normal weight, overweight (BMI z-scores above 1 SD) and obese (BMI z-scores above 2 SD). The study protocol was approved by the International Review Board of Faculty of Medicine, Chulalongkorn University, Bangkok, Thailand (IRB 237/64).

### 2.1. Measurement of Bisphenols (BPA, BPF, BPS) in Urine

The concentrations of BPA, BPF, and BPS in urine were determined using the liquid chromatography and tandem mass spectrometry (LC-MSMS) technique. For the preparation of the urine sample, each 1 mL urine sample was centrifuged at 3000 rpm for 10 min. Then, 500 µL of the urine sample was hydrolyzed by adding 100 µL of ammonium acetate and 20 µL (2000 units) of β-glucuronidase enzyme (Sigma-Aldrich, St. Louis, MO, USA). An exoglycosidase that could catalyze the breakdown of complex carbohydrates is the β -glucuronidase enzyme. It transformed conjugated bilirubin in humans into the unconjugated form, enabling bilirubin to be reabsorbable. Additionally, as demonstrated at https://www.sigmaaldrich.com/TH/en/product/sigma/g1512 (accessed on 5 September 2023), it could be employed for the hydrolysis of glucuronide conjugates in urine metabolite analysis with the % efficiency of the technique utilizing BPs-glucuronide.

The mixture was vortexed, followed by incubation at 37 °C for 3 h. Subsequently, the samples were purified by protein precipitation using 500 µL of acetonitrile purchased from JT Baker. Bisphenol A-d16, d7 BPF, and d8 BPS (Sigma-Aldrich) were used as internal standards. The samples were vortexed again, centrifuged at 3000 rpm for 10 min, and then subjected to LC-MSMS analysis. Bisphenol levels were determined using an Acquity UPLC^®^ system (Waters, Chicago, IL, USA) coupled with a XEVO-TQS mass spectrometer (Waters), with quantitation limits (LOQ) of 0.50, 0.01, and 0.01 ng/mL for BPA, BPF, and BPS, respectively. The limit of quantitation (LOQ) is the smallest quantity of the measurthat can be quantified in a sample under specified experimental conditions with stated, acceptable accuracy and acceptable precision. By comparing measured signals from samples with known low analyte concentrations with those of blank samples, it is possible to calculate the signal-to-noise ratio and determine the minimal concentration at which analyte quantification is reliable. The mean signal-to-noise ratio for BPA, BPS, and BPF was 10.36, 10.57, and 10.30, respectively, compared to a usual signal-to-noise ratio of 10. In this investigation, the LOQ for BPA, BPS, and BPF were 0.5, 0.01, and 0.01, respectively. In this study, artificial urine was used as the control sample, to create the calibration curves, and to assess participant BPA, BPS, and BPF amounts. The average RSD recoveries for BPA, BPS, and BPF were determined to be 97.9 ± 2.5, 101.2 ± 9.4, and 100.8 ± 4.5, respectively.

### 2.2. Urinary Creatinine and Specific Gravity

Urine creatinine (urine Cr) was analyzed using an enzymatic method with Alinity c Creatinine Reagent Kit 08P01 (Crea Enz) (Abbott Laboratories, Jinan, China). Urine specific gravity (SG) was measured using the urine specific gravity refractometer (MASTER-URC/NM, Atago, Japan) with the SG scale range of 1.000 to 1.050, a refractive index range of (nD) 1.333 to 1.356, a minimum SG scale of 0.001, and a minimum refractive index of 0.001. 

To adjust for urine dilutional, urinary bisphenol concentration in ng/mL was either adjusted to Cr (mcg/g creatinine) or by SG [25]. 

### 2.3. Estimated Daily Intake (EDI) of Bisphenols

Estimated daily intake (EDI) of bisphenols analog was calculated as follows [26].

$$EDI=UC\times {CE}_{smoothed}\times \frac{1}{F}\times \frac{1}{BW}$$

EDI; estimated daily intake of bisphenol (mcg/kg/day);

UC; creatinine-adjusted urinary concentrations of BPA, BPF, or BPS (mcg/g creatinine); ${CE}_{smoothed}$; the 24 h urinary creatinine excretion.

For the children, ${CE}_{smoothed}$ corresponded to age- and sex-specific average creatinine excretion levels based on data provided by Remer et al. (2002) [27]:

- Boys aged 6 to 8 years (0.49 g/day) or 9 to 13 years (0.76 g/day);
- Girls aged 6 to 8 years (0.45 g/day) or 9 to 13 years (0.72 g/day).

F; the rate of urinary excretion of bisphenols after oral exposure; The F for BPA, BPF and BPS was close to 100%, 100% [13] and 70% [14], respectively. 

### 2.4. Statistical Analysis

Characteristics of the study population were presented as mean (SD), median (IQR), and percentages. Bisphenol detection rates and concentration were compared between age, sex, and BMI groups using chi-square tests. Urine bisphenol concentration between groups was compared using a two-sample independent *t*-test. Generalized Estimating Equations (GEE) with a linear model identified factors associated with SG-adjusted bisphenol concentration, BPS/BPA ratio, and BPF/BPA ratio. Multivariate models adjusted for covariates with *p*-values < 0.1 in univariate models. Analyses were performed with STATA version 13.1 (StataCorp, College Station, TX, USA). Statistical significance was set at *p* < 0.05.

## 3. Results

### 3.1. Characteristics of the Participants

The average age of the participating children was 9.4 ± 1.7 years, with 52% being boys (Table 1). More than half of the participants had a normal BMI, had caregivers with a high school or equivalent level of education, and came from families with low monthly income.

### 3.2. Bisphenol Exposure

In the children’s urine, BPA (79.6%) was most frequently detected, followed by BPF (31.0%), and BPS (16.8%). Of all participants, 286 children provide two morning voids, and the detection rates for urinary bisphenols in each individual significant varied between the two samples (Table 2).

Among the three analytes, BPA had the highest concentration (mean GM 1.41 ng/mL), followed by BPS (mean GM 0.014 ng/mL), and BPF (mean GM 0.013 ng/mL) (Table 3). The ratio (95% CI) of BPF/BPA was 0.009 (0.008–0.010), and the BPS/BPA ratio was 0.010 (0.009–0.012). 

The estimated daily intake (EDI) derived from Cr-adjusted urine bisphenol concentrations was highest for BPA, with the 95th percentile value of 0.114 mcg/kg/day, followed by BPS with 0.003 mcg/kg/day, and BPF with 0.001 mcg/kg/day. None of these EDI exceeded the tolerable daily intake (TDI) values established by the European Food Safety Authority (EFSA) in 2015 (Table 4). 

### 3.3. Factors Correlated with Urinary Bisphenol Concentrations

Urinary BPA, BPF, and BPS concentrations were not different by obesity status and gender (Table 5). Mean urine BPA, BPF, and BPS concentrations were higher among younger children (aged < 10 years) compared to the older age group (age ≥ 10 years) (*p* < 0.05). Specifically, GMs were 1.31 times higher for BPA, 1.46 times higher for BPF, and 1.62 times higher for BPS (Table 6). Children who consumed food from plastic containers for a minimum of two days each week exhibited notably elevated bisphenol levels in comparison to those with limited exposure. Specifically, the GMs were 1.44 (1.16–1.78) times higher for BPF and 1.40 (1.05–1.87) times higher for BPS. However, when subjected to a multivariate analysis, none of the exposure variables maintained their significance. 

## 4. Discussion

In 2015, Thailand introduced regulations regarding the safety of milk bottles and containers used to feed infants and young children with the goal of reducing health concerns related to exposure to BPA. However, the current study indicates a higher BPA detection rate in urine samples from Thai children (79.6%) compared to a previous study conducted 6 years ago (during 2013–2014), which reported a BPA detection rate of 75.3% [23]. These findings suggest that despite the regulations on the use of BPA in Thailand, current exposure to BPA remains prevalent among Thai children and adolescents. Furthermore, the detection rates for BPF and BPS in our study are considerably lower than those for BPA. The very low concentration ratios of BPF/BPA and BPS/BPA in each urine sample also indicate that children are primarily exposed to BPA compared to BPF and BPS. However, there is a lack of previous data available to compare BPF and BPS levels in Thai children.

Analysis of two sets of urine samples collected with a one-week interval revealed a significant variation in the detection rate and urinary concentration of BPA and BPS, which can be attributed to variations in exposure to bisphenol analogs in children over time. The GM of urinary BPA concentration in our study (1.41 ng/mL) exceeded the levels observed in most studies on BPA exposure in children in Belgium, Japan, China, and the United States from 2013 to 2018, which reported average urinary BPA concentrations ranging from 0.25 to 1.05 ng/mL [21,22,28,29]. However, studies conducted in Brazil and the EU from 2011 to 2013 [30,31] showed higher urinary levels of BPA than in our study, with a range of 1.66 to 1.96 ng/mL. Furthermore, urinary concentrations of BPF and BPS in our current study of 0.013 ng/mL and 0.014 ng/mL, respectively, are significantly lower than those reported in almost all previous studies in other countries, which documented BPF levels ranging from 0.07 to 0.32 ng/mL and BPS levels ranging from 0.03 to 0.29 ng/mL. The variability in bisphenol concentration observed in different studies can be attributed to several factors. Different regions and populations may have varying exposure levels due to differences in consumption patterns, dietary habits, and environmental conditions. Time period and regulation changes resulted in shifts in manufacturing practices and consumer behavior. Studies conducted in different years might capture different phases of exposure. Moreover, variations in laboratory methods and detection limits can lead to differing measurements between studies. 

Urine bisphenol concentrations were associated with age of the children. Specifically, younger age of less than 10 years was associated with 1.3- to 1.6-fold higher concentrations of urine bisphenol analogs compared to older children. This reflects a high exposure to bisphenols in young children, which is similar to the findings of the NHANES study conducted in 2013–2014 [21], where children under 11 years of age had significantly higher median levels of urinary BPA compared to adolescents (GM 1.34 mcg/L vs. GM 1.14 mcg/L). Tait S et al. from Italy also demonstrated that urinary BPA concentrations were higher in children aged 4 to 6 years compared to those aged 7 to 10 years and 11 to 14 years [24]. A human biomonitoring study from Australia consistently showed that urine BPA levels in young children were significantly higher than in adults [26]. These findings may be explained by the hypothesis that children consume more of these chemicals than adults due to a higher amount of bisphenol-contaminated food consumption and through probable differences in how these compounds are absorbed, distributed, metabolized, and excreted between children and adults [21]. Another explanation is the difference in the environments and lifestyles of the two groups. Children are found to more exposed to bisphenol analogs from inhalation and ingestion of house dust when they spend more time at home than adults [10]. 

Regarding gender, our results found no distinction between the GM of Cr-adjusted urine bisphenol concentration in boys and girls. This is consistent with the results of several studies focused on bisphenol exposure in children [21,24,32], suggesting that children of both sexes have comparable levels of bisphenol exposure. Previous cross-sectional studies found correlation between urinary concentration of BPA with obesity and BMI in children [33,34,35], which may be explained by the promotion of preadipocyte differentiation by the BPA effect [36]. However, it remains unclear whether obese children consume more BPA. On the contrary, we found no association between obesity and urinary BPA concentration. This lack of association may be attributed to the small number of participants, which could limit the statistical power of the study.

In addition to age, other behavioral risk factors associated with high bisphenol exposure included the consumption of food stored in plastic containers at least twice a week, which increases the risk of having high Cr-adjusted urinary BPF and BPS levels, implying that plastic containers used for food storage are potential sources of bisphenol exposure in Thai children. Bisphenols are commonly used in the production of certain food packaging materials. When food or beverages contact with these packages, there is a possibility of bisphenol leaching into the food or drink, especially under certain conditions such as high temperatures or acidic environments [37]. Our results indicate that, in addition to BPA-free products, consideration of BPF-free and BPS-free plastic containers may also be necessary. Recent study found that BPA, BPF, and BPS were frequently detected in house dust, accounting for 9%, 38%, and 12% of the total daily bisphenol intakes, respectively [10]. Future research on Thai children may include the data collection on house dust ingestion, as it was not included in this study.

When comparing the EDI of BPA and BPS in Thai children and adolescents with the TDI established by the EFSA in 2015, the EDI values for BPA are approximately 35 times below the corresponding TDI values of 4 mcg/kg of body weight per day, and the EDI values for BPS are around 1200-fold below the TDI, reflecting that there may be no immediate health concerns for this population. However, TDI represents an estimate of the amount of a contaminant that can be consumed over a lifetime without posing an appreciable risk to health, and this study provides only cross-sectional data, EDI may not reflect overall lifetime exposure to bisphenols. Moreover, regarding the most recent scientific insights on the potential risks associated with BPA exposure to human health, EFSA’s specialists have introduced a new TDI standard of 0.2 ng/kg/day (equivalent to 0.0002 mcg/kg/day) in April 2023, which is notably lower by approximately 20,000 times compared to the previous level. Due to this updated criterion, all participants in our study exceeded the new TDI threshold. Therefore, as EFSA periodically reassesses the toxicity of bisphenols and lowers the TDI threshold, continuous biomonitoring of exposure to BPA, BPF, and BPS, together with additional research on their health effects in children, is necessary.

The novelty of this research lies in its focus on Thai children, a demographic previously under-represented that has lacking a comprehensive characterization of their exposure to BPA and its analogues, BPF and BPS. The collection of the double morning voids and use of the average concentration for analysis increase the study’s representativeness. However, it is important to acknowledge its limitations. First, this study was conducted in a single center, which can affect the generalizability of the findings to the general population. Second, the causal relationship and the mechanism that underlie the association were not established because of the nature of the cross-sectional study. Lastly, the EDI of bisphenols was derived from two single spot urine samples, rather than 24 h urine collection, which could introduce some measurement uncertainty.

In conclusion, this comprehensive study of urine bisphenol metabolites provides insight into the predominant exposure to BPA, followed by BPF and BPS, among Thai children and adolescents during the period 2019–2020, with BPA showing the highest concentration in urine. Significant associations between Cr-adjusted urine concentrations of BPA, BPF, and BPS and younger age indicate age-related differences in bisphenol exposure. High concentrations of BPF and BPS in urine were associated with exposure to food stored in plastic containers. Future research should focus on investigating the potential health effects of exposure to BPF and BPS in children, and measures for bisphenol-free products are still needed. 

## Figures and Tables

**Table 1:** Baseline characteristics of the participants (N = 358).

Characteristics	Value
Age (years; mean ± SD)	9.4 ±1.7
Sex, N (%)	
Boy	186 (52)
Girl	172 (48)
Age group, N (%)	
<10 years	211 (58.9)
≥10 years	147 (41.1)
Anthropometry, median (IQR)	
Body weight (kg.)	32.7 (25.1–41.4)
Height (cm.)	134.2 (126–144)
Waist circumference (cm.)	60.5 (55–70.5)
BMI (kg/m^2^), median (IQR)	17.3 (15.4–20.9)
Underweight, N (%)	12 (3.3)
Normal, N (%)	209 (58.9)
Overweight, N (%)	56 (15.8)
Obese, N (%)	78 (22)
Caregiver Education, N (%)	
<High school	53 (18.3)
<High school or equivalent	159 (54.8)
≥Bachelor’s degree	78 (26.9)
Family Income (USD/month), n (%)	
<850	155 (54.2)
850–1400	111 (38.8)
>1400	20 (7)
Residence, N (%)	
Apartment/condominium/townhouse/shop house/other	296 (82.7)
Detached house	62 (17.3)

n: number of participants in subgroup; BMI: body mass index.

**Table 2:** Bisphenol detection rate in urine, N (%).

	Detection Rate from First Urine Samples (N = 358)	Participant Who Provides Both Urine Samples (N = 286)		*p*
		Detection Rate from First Urine Samples	Detection Rate from Second Urine Samples	
**BPA**	285 (79.6)	235 (82.2)	173 (60.5)	<0.001
**BPF**	111 (31.0)	98 (34.3)	60 (21.0)	<0.001
**BPS**	60 (16.8)	49 (17.1)	14 (4.9)	<0.001

The *p*-value was evaluated using McNemar’s test.

**Table 3:** Values of urine bisphenol levels of Thai children and adolescents.

Analyte	10th	25th	50th	75th	90th	GM	95%CI of GM
**Unadjusted urine bisphenol concentration (ng/mL)**							
**BPA**							
1st sample	0.5	0.607	1.384	2.766	4.924	1.487 *	1.348–1.641
2nd sample	0.5	0.5	0.7005	1.425	2.61	0.934 *	0.847–1.03
Mean	0.5085	0.754	1.296	2.124	3.7475	1.414	1.278–1.565
**BPF**							
1st sample	0.01	0.01	0.01	0.012	0.022	0.013	0.012–0.013
2nd sample	0.01	0.01	0.01	0.01	0.019	0.012	0.011–0.012
Mean	0.01	0.01	0.01	0.014	0.022	0.013	0.012–0.014
**BPS**							
1st sample	0.01	0.01	0.01	0.01	0.067	0.015 *	0.013–0.017
2nd sample	0.01	0.01	0.01	0.01	0.01	0.011 *	0.01–0.012
Mean	0.01	0.01	0.01	0.01	0.0485	0.014	0.013–0.016
**Cr-adjusted urine bisphenol concentration (mcg/g Cr)**							
**BPA**							
1st sample	0.560	0.927	1.446	2.446	4.920	1.586 *	1.442–1.744
2nd sample	0.426	0.634	0.950	1.662	2.886	1.094 *	0.985–1.215
Mean	0.591	0.864	1.291	1.958	3.138	1.389	1.262–1.529
**BPF**							
1st sample	0.005	0.007	0.011	0.021	0.047	0.014	0.012–0.015
2nd sample	0.006	0.008	0.012	0.019	0.042	0.014	0.013–0.015
Mean	0.006	0.008	0.012	0.017	0.030	0.013	0.012–0.014
**BPS**							
1st sample	0.005	0.007	0.010	0.023	0.104	0.016	0.014–0.018
2nd sample	0.005	0.007	0.010	0.018	0.044	0.013	0.012–0.015
Mean	0.006	0.007	0.011	0.018	0.048	0.014	0.013–0.016
**SG-adjusted urine bisphenol concentration (ng/mL)**							
**BPA**							
1st sample	0.5	0.607	1.384	2.766	4.924	1.487 *	1.348–1.641
2nd sample	0.500	0.500	0.691	1.425	2.610	0.932 *	0.845–1.029
Mean	0.601	0.829	1.348	2.176	3.729	1.471	1.331–1.625
**BPF**							
1st sample	0.01	0.01	0.01	0.012	0.022	0.013	0.012–0.013
2nd sample	0.010	0.010	0.010	0.010	0.019	0.012	0.011–0.013
Mean	0.008	0.009	0.012	0.016	0.026	0.014	0.013–0.015
**BPS**							
1st sample	0.01	0.01	0.01	0.01	0.067	0.015 *	0.013–0.017
2nd sample	0.010	0.010	0.010	0.010	0.010	0.011 *	0.01–0.012
Mean	0.008	0.009	0.011	0.016	0.044	0.015	0.013–0.017
**Bisphenol concentration ratio**							
**BPF/BPA**							
1st sample	0.002	0.004	0.010	0.020	0.033	0.010	0.009–0.012
2nd sample	0.004	0.008	0.017	0.020	0.020	0.013	0.011–0.014
Mean	0.003	0.006	0.010	0.016	0.021	0.009	0.008–0.01
**BPS/BPA**							
1st sample	0.002	0.004	0.009	0.020	0.020	0.009	0.008–0.010
2nd sample	0.004	0.007	0.015	0.020	0.020	0.012	0.011–0.014
Mean	0.003	0.005	0.010	0.019	0.040	0.01	0.009–0.012

GM: geometric mean; CI: confidence interval; * *p* < 0.001 from pair *t*-test.

**Table 4:** Estimated daily intake of bisphenol (mcg/kg/day).

Analyte	25th	50th	75th	95th	Regulation (TDI)	N (%) > TDI
BPA	0.0125	0.0213	0.0448	0.1136	4	0 (0%)
BPF	0.0001	0.0002	0.0004	0.0011	N/A	N/A
BPS	0.0002	0.0003	0.0006	0.0034	4.4	0 (0%)

TDI: tolerable daily intake (mcg/kg/day), an estimate of the amount of a contaminant, which can be consumed over a lifetime without presenting an appreciable risk to health, value provided by EFSA in 2015. N/A: not available.

**Table 5:** Comparison of GM of Cr-adjusted urine bisphenol concentration between different groups of participants.

Parameter	Group	BPA	*p*	BPF	*p*	BPS	*p*
**Obesity**	Not Obese (N = 226)	1.341 (1.223–1.471)	0.165	0.013 (0.012–0.015)	0.107	0.046 (0.011–0.081)	0.727
	Obese (N = 60)	1.584 (1.171–2.143)		0.011 (0.01–0.013)		0.059 (−0.013–0.13)	
**Sex**	Boy (N = 153)	1.41 (1.209–1.643)	0.748	0.013 (0.011–0.014)	0.391	0.052 (0.001–0.102)	0.842
	Girl (N = 133)	1.366 (1.225–1.523)		0.013 (0.012–0.015)		0.045 (0.012–0.078)	
**Age**	Age < 10 years (N = 182)	1.533 (1.347–1.745)	**0.007**	0.015 (0.013–0.017)	**<0.001**	0.046 (0.021–0.071)	**<0.001**
	Age ≥ 10 years (N = 104)	1.169 (1.026–1.332)		0.01 (0.009–0.012)		0.053 (−0.021–0.127)	

The *p*-value was evaluated by two sample independent *t*-test.

**Table 6:** Factors associated with urinary concentration of bisphenols.

Variable	Cr-Adjusted BPA		Cr-Adjusted BPF		Cr-Adjusted BPS		BPF/BPA Ratio		BPS/BPA Ratio	
	GMR (95%CI)	*p* *	GMR (95%CI)	*p* *	GMR (95%CI)	*p* *	GMR (95%CI)	*p* *	GMR (95%CI)	*p* *
**Age < 10 vs. ≥ 10 years**	1.31 (1.07–1.6)	0.007	1.46 (1.22–1.73)	0.000	1.62 (1.26–2.08)	0.000	1.12 (0.87–1.42)	0.391	1.23 (0.91–1.67)	0.167
**Obese/yes**	1.19 (0.93–1.49)	0.165	0.84 (0.68–1.04)	0.107	0.95 (0.7–1.28)	0.727	0.71 (0.54–0.94)	0.018	0.8 (0.57–1.14)	0.223
**Food with plastic container ^a^**	1.2 (0.99–1.45)	0.061	1.44 (1.16–1.78)	0.001	1.40 (1.05–1.87)	0.024	1.2 (0.96–1.5)	0.108	1.17 (0.87–1.58)	0.31
**Water in plastic bottle ^a^**	1.14 (0.92–1.42)	0.229	1.06 (0.87–1.28)	0.564	1.11 (0.84–1.46)	0.456	0.93 (0.71–1.21)	0.576	0.97 (0.7–1.35)	0.867
**Toothpaste ^b^**	1.09 (0.85–1.4)	0.485	1.13 (0.9–1.4)	0.306	1.09 (0.79–1.51)	0.578	1.03 (0.76–1.39)	0.850	1 (0.69–1.46)	0.995
**Soap ^b^**	1.11 (0.86–1.43)	0.434	1.06 (0.84–1.34)	0.641	1.03 (0.74–1.42)	0.872	0.95 (0.7–1.3)	0.767	0.92 (0.63–1.36)	0.702
**Face/body lotion ^b^**	1 (0.83–1.22)	0.977	1.02 (0.85–1.21)	0.866	0.9 (0.7–1.15)	0.418	1.01 (0.8–1.28)	0.919	0.9 (0.68–1.21)	0.481
**Dishwashing liquid ^b^**	1.03 (0.84–1.25)	0.768	1.05 (0.89–1.26)	0.551	0.84 (0.66–1.07)	0.168	1.02 (0.81–1.3)	0.840	0.82 (0.61–1.09)	0.173

GMR: Geometric mean ratio. ^a^ ≥2 days per week vs. ≤Once a month. ^b^ Yes, within 24 h vs. >24 h. * *p* < 0.05 indicate statistical significance from univariate analysis.

## Data Availability

The data presented in this study are available on request from the corresponding author. The data are not publicly available due to ethical issue.
